# Association between Variants in the *OCA2-HERC2* Region and Blue Eye Colour in *HERC2* rs12913832 AA and AG Individuals

**DOI:** 10.3390/genes14030698

**Published:** 2023-03-11

**Authors:** Nina Mjølsnes Salvo, Jeppe Dyrberg Andersen, Kirstin Janssen, Olivia Luxford Meyer, Thomas Berg, Claus Børsting, Gunn-Hege Olsen

**Affiliations:** 1 Centre for Forensic Genetics, Department of Medical Biology, Faculty of Health Sciences, UiT The Arctic University of Norway, 9037 Tromsø, Norway; 2 Section of Forensic Genetics, Department of Forensic Medicine, Faculty of Health and Medical Sciences, University of Copenhagen, 2100 Copenhagen, Denmark

**Keywords:** forensic genetics, DNA phenotyping, massively parallel sequencing, *OCA2-HERC2*, eye colour, pigmentation

## Abstract

The *OCA2-HERC2* region is strongly associated with human pigmentation, especially eye colour. The *HERC2* SNP rs12913832 is currently the best-known predictor for blue and brown eye colour. However, in a previous study we found that 43 of 166 Norwegians with the brown eye colour genotype rs12913832:AA or AG, did not have the expected brown eye colour. In this study, we carried out massively parallel sequencing of a ~500 kbp *HERC2-OCA2* region in 94 rs12913832:AA and AG Norwegians (43 blue-eyed and 51 brown-eyed) to search for novel blue eye colour variants. The new candidate variants were subsequently typed in a Norwegian biobank population (total *n* = 519) for population specific association analysis. We identified five new variants, rs74409036:A, rs78544415:T, rs72714116:T, rs191109490:C and rs551217952:C, to be the most promising candidates for explaining blue eye colour in individuals with the rs12913832:AA and AG genotype. Additionally, we confirmed the association of the missense variants rs74653330:T and rs121918166:T with blue eye colour, and observed lighter skin colour in rs74653330:T individuals. In total, 37 (86%) of the 43 blue-eyed rs12913832:AA and AG Norwegians could potentially be explained by these seven variants, and we suggest including them in future prediction models.

## 1. Introduction

Many pigmentation genes have been extensively studied to explain human eye colour variation [1]. Oculocutaneous albinism type 2 (*OCA2*) and its neighbouring gene the HECT domain and RCC1-like domain 2 (*HERC2*) are of special interest because of their strong genetic influence on human pigmentation, especially eye colour variation [1,2]. *OCA2* expression is regulated by the intronic SNP rs12913832, which is situated in a conserved enhancer region in *HERC2* [3,4]. The ancestral A-allele in rs12913832 allows transcription factors to modulate long-range chromatin looping that leads to contact between the *OCA2* promotor and the enhancer. This enhances *OCA2* expression and thereby, melanin production. In contrast, the derived G-allele reduces chromatin looping and thereby, *OCA2* expression and melanin production [3]. European populations have the largest variation in eye colour, and recent selection for the G-allele in Europeans has been documented [5]. The strong association between genotypes of rs12913832 and blue and brown eye colour allows accurate eye colour prediction, which has an important application in forensic genetics [4,6,7]. According to the dominant hypothesis, the genotypes rs12913832:AA or AG lead to brown eye colour, whereas the genotype rs12913832:GG is found in individuals with blue eye colour. This is correct for most individuals, but discrepancies in European populations are documented, where individuals with the genotype rs12913832:AA or AG have blue eye colour, and individuals with the genotype rs12913832:GG have brown eye colour [8,9,10]. Other polymorphisms in *OCA2*, both in regulatory and coding regions, have shown associations with eye colour, albeit with minor additive effects [11,12]. The missense *OCA2* SNP rs1800407 was included in the IrisPlex prediction tool and improved the prediction accuracy of eye colour in a European population [6,13,14], whereas the missense variants rs74653330 and rs121918166 have been suggested to explain blue eye colour in Scandinavians [8]. Additionally, rs74653330 and rs121918166 are found to be associated with human skin colour variation [8,15]. In a previous study, we showed that the eye colour prediction accuracy in a Norwegian study population increased when rs74653330 and rs121918166 were included in the prediction model [16]. However, the eye colour of some blue-eyed Norwegians were still not accurately predicted, suggesting that other variants in or around *OCA2* could affect eye colour formation in individuals with the rs12913832:AA and AG genotypes. Therefore, in this study, we sequenced the *OCA2-HERC2* region in a Norwegian biobank population in order to find new candidate variants that may explain blue eye colour in individuals with the rs12913832:AA and AG genotype.

## 2. Materials and Methods

### 2.1. Study Population and Selection of Individuals for Genotyping

A Norwegian biobank population of 540 volunteers (presumably unrelated) residing in northern Norway from 2015 to 2017, was used to search for candidate variants that may explain blue eye colour [10]. The *OCA2-HERC2* region was sequenced in a selected study cohort of 94 individuals, previously genotyped as *HERC2* rs12913832 AA or AG [10]. For the sequenced study cohort, 43 individuals were selected because they did not follow the dominant hypothesis and had blue eye colour (study group, Table 1). Additionally, 41 of the 43 individuals were previously predicted incorrectly to have brown eye colour using the IrisPlex model [10,13] (Table S1). The remaining 51 individuals had the expected brown eye colour phenotype and were used as controls (control group, Table 1). The new candidate variants were subsequently typed in the remaining Norwegian samples (*n* = 446). All samples were collected with informed consent and subsequently anonymised. The project was approved by the Faculty of Health Sciences, UiT-The Arctic University of Norway (reference number 2021/2034).

### 2.2. Eye Colour Categorisation and Quantitative Eye Colour Measurements

High resolution photographs were taken of the participants’ eyes, and categorisation of eye colours was performed in the recent study from Salvo et al. [10]. In short, nine untrained individuals were asked to intuitively assign each photograph to one of four categories: blue, intermediate-blue, intermediate-brown, or brown eyes. Herein, a two-category system (blue and brown) was used. Therefore, blue and intermediate-blue were grouped into the blue category (*n* = 43), and brown and intermediate-brown were grouped into the brown category (*n* = 51). A quantitative eye colour score (Pixel Index of the Eye (PIE)—score) was calculated for each individual’s eye photograph using the custom-made Digital Analysis of Iris Tool software (DIAT) v.1 [9]. All the photographs from individuals categorised with blue eye colour had PIE-scores ranging from −0.3 to 1, and all the photographs from individuals categorised with brown eye colour had PIE-scores from −1 to −0.07 (Table S1). Only two individuals with blue eyes and six individuals with brown eyes had PIE-scores in the overlapping region (−0.3 to −0.07). The categorised eye colour highly correlated with the PIE-scores (Spearman’s correlation coefficient: −0.85, *p* < 0.001). For further details, see Salvo et al. [10].

### 2.3. Quantitative Skin Colour Measurements

Quantitative measurements of skin colour were performed using a NCS (Natural Colour System) Colour Scan 2.0 on the inside of the upper arm on 507 individuals in the Norwegian biobank population. The output metrics consisted of L* a* b* (L* = lightness, a* = red, b* = yellow). The L* value measures skin lightness, ranging from 0 to 100, where 0 is the darkest and 100 is the lightest. For a consensus measurement, at least three measurements were made, avoiding pigmentation spots and hair.

### 2.4. Probe Design and Sequencing the OCA2-HERC2 Region

The software SureDesign (Agilent Technologies, Santa Clara, CA, USA) was used to design capture-probes for a ~500 kbp region on chromosome 15 (GRCh38, chr15: 27,754,870–28,254,863) for SureSelectXT HS2 Target Enrichment System (Agilent Technologies, Santa Clara, CA, USA). The design included 15,627 probes targeting 427,954 bp. The library preparation was carried out according to the SureSelextXT HS2 DNA System protocol, version D0. All samples were sequenced on an Illumina MiSeq (Illumina, San Diego, CA, USA) according to the manufacturer’s instructions with paired-end sequencing (2 × 150 bp) using the MiSeq Reagent Kit V2 (300 cycles).

### 2.5. Analysis of Sequencing Data

The sequencing output was automatically converted to FASTQ files with the MiSeq Reporter Software. Agilent’s molecular barcode (MBC) sequences were extracted using the Agilent Genomics NextGen Toolkit (AGeNT), and the FASTQ files were trimmed using AdapterRemoval version 2.1.3 [17,18] with a minimum read length of 30 bp and Phred quality score of Q = 30. The files were subsequently aligned to the human reference sequence assembly GRCh38/hg38 with the Burrows–Wheeler Aligner, BWA-MEM algorithm [19,20]. Sequence alignment map (SAM) files were converted into binary alignment map (BAM) files using SAMtools [21]. The Genome Analysis Toolkit (GATK) with HaplotypeCaller version 4.0.0.0 [22] was used to create Variant Call Format (VCF) files. Variants located in the regions of interest were extracted using BEDTools version 2.22.1 [23]. Genotypes were accepted if the read depth was ≥10 and the heterozygote balance (Hb = read depth of allele/read depth of nucleotide position) was 0.20 < Hb < 0.80.

The *fisher.test* and *Kruskal.test* commands in R: A Language and Environment for Statistical Computing version 4.2.1 (R Foundation for Statistical Computing, Vienna, Austria) [24] were used to perform Fisher’s exact test and Kruskal–Wallis test of the statistical association between genotype data, and eye colour categories and PIE-scores, respectively. Mann–Whitney tests for two independent samples and linear regression analysis were performed using the Real Statistics Resource Pack software (release 6.3; copyright (2013–2021) Charles Zaiontz, www.real-statistics.com, accessed on 16 January 2022). Hardy–Weinberg equilibrium (HWE) and pairwise r^2^ values for linkage disequilibrium (LD) testing were calculated using Haploview 4.2 [25]. For predictions of regulatory elements, the positions of the candidate variants were overlapped with datasets from the SCREEN: Search Candidate cis-Regulatory Elements by ENCODE platform, Registry of cCREs V3 [26]. Haplotypes were estimated using PHASE version 2.1 (University of Washington, Seattle, WA, USA) [27,28], using default settings.

### 2.6. Variant Typing

Eight candidate variants for blue eye colour, rs551217952, rs543971307, rs191109490, rs78544415, rs74409036, rs62008729, rs72714116 and rs12913832, were typed in the remaining 446 individuals in the Norwegian biobank population (total *n* = 540) using the SNaPshot^TM^ multiplex system (Thermo Fisher Scientific, Waltham, MA, USA). The SNPs rs543971307 and rs191109490 were in complete LD. Qiagen Multiplex PCR kit (Qiagen, Hilden, Germany) was used to amplify DNA in one multiplex reaction in a final volume of 10 µL. The following PCR conditions were used: 95 °C for 15 min, 35 cycles of 94 °C for 30 s, 58 °C for 30 s and 72 °C for 30 s, and final extension at 72 °C for 10 min. A total of 5 µL amplified DNA was treated with 2 µL ExoSAP-ITTM PCR product clean-up reagent (Thermo Fisher Scientific, Waltham, MA, USA) at 37 °C for 60 min and 75 °C for 15 min. Single base extension (SBE) was performed with 1 µL purified PCR product, 1 µL MilliQ-water, 2 µL SNaPshot™ Multiplex Ready Reaction Mix (Thermo Fisher Scientific, Waltham, MA, USA) and 1 µL SBE primer mix (Table S2). The following cycle conditions were used: 30 cycles of 96 °C for 10 s, 55 °C for 5 s and 60 °C for 30 s. The SBE PCR products were then treated with 1 µL SAP (Thermo Fisher Scientific, Waltham, MA, USA) at 37 °C for 30 min and 75 °C for 15 min. Separation and detection of treated SBE products were carried out on a 3500xL Genetic Analyzer (Thermo Fisher Scientific, Waltham, MA, USA) using FragmentAnalysis with POP-4™ polymer, a 36 cm capillary and Dye Set E5. The analysis was performed with 1 µL SAP-treated SBE products, 20 µL Hi-Di formamide and GeneScan™-120 LIZ^®^ Size Standard (200:1). The results were analysed with GeneMapper^®^ ID-X v.1.5 (Thermo Fisher Scientific, Waltham, MA, USA), with a minimum peak height threshold of 50 RFU. In total, 425 individuals were successfully typed for all variants and were, together with the 94 sequenced individuals, used for population-specific analysis (total *n* = 519).

## 3. Results

### 3.1. Sequencing of the HERC2-OCA2 Region and Discovery of Candidate Blue Eye Colour Variants

Approximately 500 kbp of the *HERC2-OCA2* region was sequenced in a study cohort of 94 individuals (43 in study group and 51 in control group). Across the target region, the median read depth ranged from 116 to 498 reads. In the captured 427,954 bp, a total of 2571 variants were identified after excluding 16 variant positions with less than 10 reads in more than 5% of the samples. Of the 2571 variants, 146 showed significant deviations from HWE (*p*-value < 0.05), and many variants were in strong or complete LD (pairwise r^2^ ≥ 0.8). A total of 419 independent variants were identified using the Tagger function in Haploview. Among the rs12913832 AA individuals, 24 variants were statistically significantly associated with both the PIE-score and eye colour categories (Kruskal–Wallis test and Fisher’s exact test, raw *p*-values < 0.05; Table S3). Among the rs12913832 AG individuals, 47 variants were statistically significantly associated with eye colour using both tests (Table S3). None of the variants were statistically associated with eye colour after Bonferroni correction for multiple testing (*p*-value < 0.00012 with m = 419 independent loci).

Six missense *OCA2* variants were observed in the study cohort (rs1800414, rs74653330, rs121918166, rs1800407, rs1800401 and rs33929465). The SNPs rs74653330:T and rs121918166:T were observed in 24 individuals (56%) from the study group, whereas they were only observed in five individuals (9.8%) from the control group. These variants were statistically significantly associated with both quantitative and categorical eye colour (Kruskal–Wallis/Mann–Whitney test and Fisher’s exact test, raw *p*-values < 0.05). Thus, rs74653330:T and rs121918166:T could potentially explain blue eye colour (Figure S1). These two variants were previously typed in the same biobank population [16]. The other four missense variants (rs1800414, rs1800407, rs1800401 and rs33929465) were not associated with blue eye colour.

**Figure 1:**
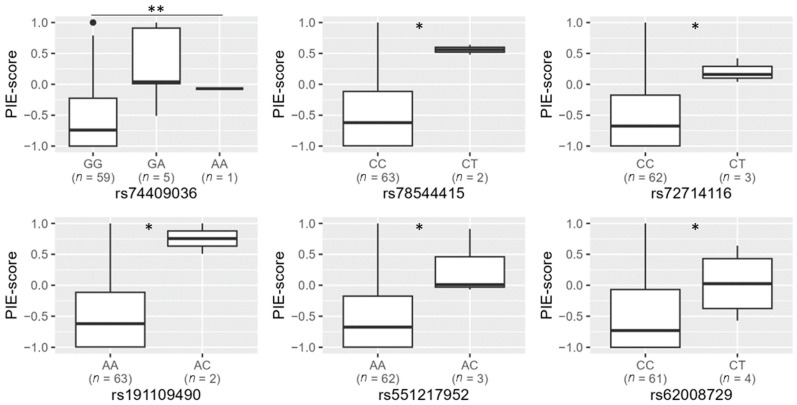
Genotypes and PIE-scores for the candidate blue eye colour variants rs74409036, rs78544415, rs72714116, rs191109490, rs551217952 and rs62008729 in 65 rs12913832 AA and AG individuals that did not have the missense variants rs74653330:T and rs121918166:T. Statistical significance (Mann–Whitney test): * *p* < 0.05, ** *p* < 0.01.

When searching among the individuals who did not carry the two missense variants, rs74653330:T and rs121918166:T (*n* = 65), we selected the six *OCA2-HERC2* candidate variants rs74409036, rs78544415, rs72714116, rs191109490, rs551217952 and rs62008729 as the most promising candidates to explain blue eye colour (Table 2). These variants had a significant association with the PIE-score (Mann–Whitney test, raw *p*-value < 0.05), and the variant (minor) alleles were primarily observed in the study group and not in the control group (Figure 1 and Table 2). For population-specific association analysis, these candidate variants were typed in the remaining samples of the Norwegian biobank population.

### 3.2. Candidate Blue Eye Colour Variants and Their Association with Eye Colour in the Norwegian Population

The six candidate variants were all intronic, either in *OCA2* or *HERC2* (Figure S2). Only one variant, rs191109490, was predicted by SCREEN [26] to be in a candidate cis-regulatory element (cCRE) in foreskin melanocytes (EH38E1749549). Interestingly, the locus is located in *HERC2*, only 69bp from rs12913832 (r^2^ = 0). Notably, rs191109490 was in complete LD with rs543971307 (r^2^ = 1), which was also found to be in the cCRE in *HERC2*, close to rs12913832.

When typed in the Norwegian biobank population (*n* = 519), the six candidate variants had low frequencies (0.002–0.082) and were mainly observed in blue eyed individuals (Table 3 and Figure S3). Of the six variants, the SNP rs62008729:T showed the highest frequency of 0.082 (Table 1). However, five of eleven (45%) rs12913832 AG individuals carrying this variant had brown eyes (Figure S3). Therefore, this SNP was excluded from further analysis.

**Figure 2:**
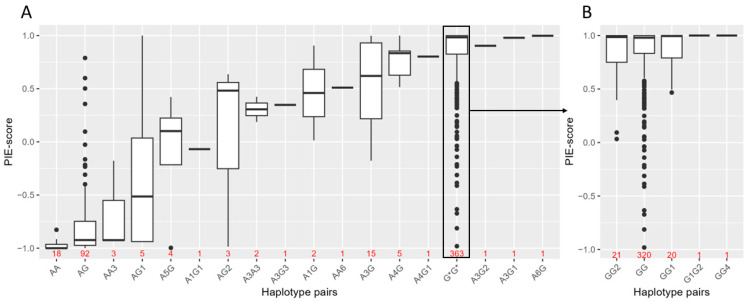
Estimated haplotype pairs by PHASE and PIE-scores in the Norwegian biobank population (*n* = 519) comprised of the haplotypes in Table 4. (**A**) Shows rs12913832 AA and AG haplotype pairs. All rs12913832 GG haplotype pairs are grouped together as G*G* and are shown in more detail in (**B**) (*n* = 363). G* = rs12913832:G genotype regardless of haplotype. The number of individuals carrying the haplotype pair is indicated in red.

For statistical association analysis with eye colour variation in the Norwegian biobank population, haplotypes were estimated based on the genotypes of the five candidate variants, rs74409036:A, rs78544415:T, rs72714116:T, rs191109490:C and rs551217952:C, and with the missense variants, rs74653330:T and rs121918166:T. In total, 11 haplotypes were estimated (Table 4). The best estimated haplotype pairs (by PHASE) and PIE-scores for all 519 individuals are shown in Figure 2. Despite some of the haplotypes being observed in only a few individuals, the total correlation of the haplotypes to the PIE-score was 0.8 (linear regression, adjusted R^2^ = 0.80). All haplotype pairs, except AA, AG, AA3 and AG1, had a median PIE-score higher than −0.3, indicating blue eye colour (Figure 2A). The median PIE-scores of the haplotype pair AA and AG were −1 (Q1 = −1 and Q3 = −0.95) and −0.92 (Q1 = −0.97 and Q3 = −0.74), respectively. This indicates that most rs12913832 AA and AG individuals with blue eye colour did not carry one of these two haplotype pairs. The haplotype AG1 had the largest variation, with PIE-score ranging from −0.94 (brown) to 1 (blue), but this haplotype was only observed in five individuals. The haplotype A5G had a smaller variation (PIE-score ranging from −1 to 0.42), but the median PIE-score was 0.10. All, except one brown-eyed individual carrying this haplotype, had blue eyes with a pupillary ring of brown, resulting in perception of green elements. Interestingly, AA6 and A6G, with the rs191109490:C variant that was predicted to be in a cCRE, both showed high PIE-scores of 0.51 (blue) and 1 (blue), respectively. All rs12913832 GG haplotype pairs had median PIE-scores of 0.98 to 1 (blue) (Figure 2B).

### 3.3. HERC2-OCA2 Haplotypes and Their Association with Skin Colour in the Norwegian Population

**Figure 3:**
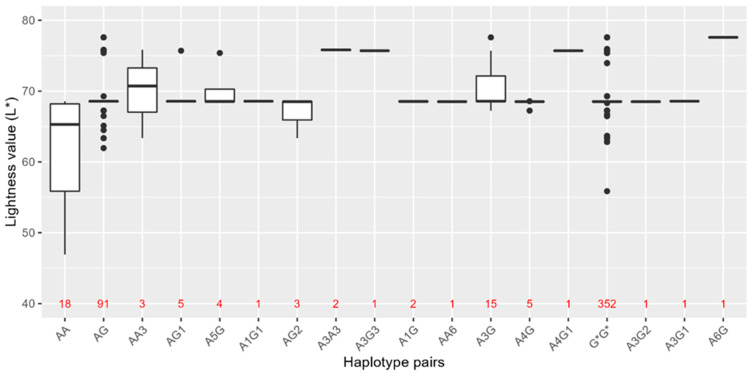
Estimated haplotype pairs by PHASE and lightness value (L*) of skin colour in the Norwegian biobank population (*n* = 507), comprised of the rs12913832 AA and AG haplotype pairs (Table 4). All rs12913832 GG haplotype pairs are grouped together as G*G*. G* = rs12913832:G genotype regardless of haplotype. The number of individuals carrying each haplotype pair is indicated in red.

Because SNPs in *HERC2-OCA2* are also associated with skin pigmentation, the haplotypes in Table 4 were assessed for association with skin shade, from dark to light, in the Norwegian biobank population (Figure 3). The lightness values (L*) of 507 individuals were measured and used as a metric for skin colour. The L* value in the population varied from 46.92 to 77.58, with a median of 68.51. No overall significant associations were observed between the haplotypes and L* values. However, a trend of lighter skin colour was observed in individuals with rs74653330:T on a background of rs12913832:AA and AG genotypes. In total, 21 (91%) of 23 individuals with rs74653330:T (Haplotypes A3 and G3) had L* values higher or equal to the median skin colour in the population (L* = 68.51), indicating light skin colour. Furthermore, individuals with A3G had significantly lighter skin colour than the rest of the population (raw *p*-value < 0.02).

## 4. Discussion

In the present study, we sequenced a ~500 kbp region of *OCA2-HERC2* to search for candidate blue eye colour variants in individuals carrying the rs12913832:AA or AG genotype. In the Norwegian biobank population, 43 individuals (study group) did not have the expected brown eye colour based on rs12913832 [10]. Because of the strong selection pressure on *OCA2-HERC2* in blue-eyed Europeans [5], it was hypothesised that other variants in this region may have an effect on OCA2 function, either by regulating the protein expression or by altering the protein.

Assessment of the sequencing quality showed that the captured region was successfully sequenced with good quality (median read depth between samples ranged from 116 to 498 reads across the target regions). Only 16 variant positions had less than 10 reads in more than 5% of the samples and were excluded. Four of these variants were in *OCA2*, and one of them overlapped with a deletion that was previously identified in 35% (15/43) of the study samples, which could potentially explain the low read depth [29].

In addition, to confirm the association of the missense variants rs74653330 and rs121918166 with eye colour, we identified five new candidate variants (rs74409036:A, rs78544415:T, rs72714116:T, rs191109490:C and rs551217952:C) that may potentially explain blue eye colour in individuals with the rs12913832:AA and AG genotype. Independently, they were all associated with quantitative eye colour (PIE-score) in the Norwegian biobank population, and 37 (86%) of the 43 blue-eyed individuals in the study group had at least one of the seven variants (including rs74653330:T and rs121918166:T). To our knowledge, this is the first time that the variants rs74409036, rs78544415, rs72714116, rs191109490 and rs551217952 are reported to be associated with human eye colour variation. These variants have very low frequencies and are mainly observed in Europeans, and some of them also in Americans (https://gnomad.broadinstitute.org/, accessed on 8 December 2022). The SNP rs74653330:T is also moderately frequent in East Asia, where it is associated with lighter skin colour [15].

The candidate variants rs74409036, rs78544415, rs72714116, rs551217952 and rs191109490 are all intronic. With the currently available data, rs191109490 was the only variant found by SCREEN to be in a cCRE in melanocytes [26]. Interestingly, rs191109490 is contained in a distal enhancer-like signature in *HERC2* in foreskin melanocytes, indicating a regulatory effect on *OCA2* expression and thereby, pigmentation. However, the specific role of rs191109490 in the cCRE is yet to be identified. Despite rs191109490:C only being observed in two individuals (haplotype pairs: AA6 and A6G), these had higher PIE-scores (perceived as blue) than individuals with the AA and AG haplotype pairs. Notably, one individual carried the rs12913832:AA genotype and was incorrectly predicted to have brown eyes with a high probability (*p*-value = 0.96) by the IrisPlex tool (Table S1). Additionally, the individual with A6G showed lighter skin colour than most GG haplotypes, supporting the hypothesis that rs191109490:C influences *OCA2* expression.

Individuals with rs72714116:T had the same haplotype pair, A5G, and four of the five rs72714116:T individuals had PIE-scores close to the mid-scale (0.05–0.4). These individuals had intermediate-blue eye colour that might be perceived as green or hazel. As discussed in many studies, eye colour is on a continuous scale from dark brown to light blue, and perception of colour varies from person to person (e.g., [10,30,31]). The most popular prediction tool for eye colour in forensic genetics, the IrisPlex, uses three eye colour categories, blue, intermediate and brown [6,13,14]. However, prediction of intermediate eye colour has proved to be difficult because of the lack of markers associated with the intermediate eye colour and because of the individual perception of eye colour [6,10,30,31]. Thus, several authors have suggested using a two-category system (blue and brown) [16,32,33] and others a quantitative system [9,34]. Our results indicate that the variant rs72714116:T may be an example of a marker for non-blue/non-brown eye colours in the Norwegian biobank population. However, this was observed in only four individuals, and the findings needs to be replicated in more samples.

Individuals estimated with the rs74409036:A rs12913832:G haplotype (AG1) had large variations in PIE-scores, and therefore it may not be easy to predict the eye colour of AG1 individuals. However, when rs74409036:A was observed together with other variants in the haplotype pairs A1G, A3G1 and A4G1, the individuals’ PIE-scores were significantly higher (perceived as blue) than AG1 individuals. Individuals with A3G1 and A4G1 haplotypes also carried the missense mutations rs74653330:T and rs121918166:T. These two variants are associated with blue eye colour in Scandinavians [8]. We have previously demonstrated that inclusion of these variants in the EC11 prediction model led to more correct predictions in the Norwegian biobank population than with the IrisPlex model [16], which supports their importance for prediction of eye colour. Interestingly, three blue-eyed individuals in the study group had the genotype rs12913832:AA, but were predicted to have brown eye colour by the IrisPlex model with high *p*-values (0.96, 0.97 and 0.98). In addition to the one individual with rs191109490:C (already mentioned), two had the rs74653330:T variant, potentially explaining blue eye colour in all these rs12913832:AA individuals.

In the present study, we also observed lighter skin colour in individuals with the rs74653330:T rs12913832:A haplotype (haplotype pairs: AA3, A3A3, A3G3 and A3G). This is in line with previous findings that this variant is associated with skin pigmentation variation in East Asians and Scandinavians [8,15]. Andersen et al. [8] observed lighter skin colour in individuals with the rs121918166:T rs12913832:A haplotype compared to rs12913832:GG individuals. However, we did not observe this trend in the Norwegian biobank population.

Here, we demonstrate high correlation between variants in the *HERC2-OCA2* region and eye colour in the Norwegian biobank population. Their independent effect on human eye colour variation may be smaller than the *HERC2* SNP rs12913832. However, including them in prediction models improves the precision for prediction of blue-eyed individuals with rs12913832:AA and AG genotypes. It would be interesting to assess the associations in similar populations, e.g., other Scandinavian or Northern European populations. While the *HERC2-OCA2* region explains most of the blue and brown eye colour variability in Europeans, SNPs in other pigmentation genes, such as *TYR, TYRP1, SLC24A4, SLC45A2, ASIP* and *IRF4*, are also found to be associated with eye colour [35,36,37,38,39], albeit with varying population-specific effects [32,40]. We previously assessed the association between the IrisPlex SNPs (rs12913832 in *HERC2*, rs1800407 in *OCA2*, rs12896399 in *SLC24A4*, rs16891982 in *SLC45A2*, rs1393350 in *TYR* and rs12203592 in *IRF4*) and PIE-score in the Norwegian biobank population, and only observed rs16891982 in *SLC45A2* to be significantly associated with blue eye colour in rs12913832:AA and AG individuals [10]. The protein SLC45A2 might have a similar role in melanosome maturation as OCA2 [41]. Thus, *SLC45A2* may also be a target of interest to search for new blue eye colour variants. A recent GWAS identified 50 novel loci associated with eye colour, including pigmentation genes and genes involved in iris morphology and structure [42]. This demonstrates that human eye colour is a genetically highly complex trait, and the molecular mechanisms behind eye colour formation are yet to be fully understood. In the current study, we identify intronic variants to be associated with blue eye colour. Although the functional role of these variants in eye colour formation is still unknown, we suggest including them in future prediction models.

## Figures and Tables

**Table 1:** Number of rs12913832 AA and AG individuals with blue or brown eye colour selected for sequencing of the *OCA2-HERC2* region.

rs12913832	Brown (Control Group)	Blue (Study Group)
AA	21	3
AG	30	40
Total	51	43

**Table 2:** The six *OCA2-HERC2* candidate blue eye colour variants.

Candidate Variant	Chromosomal Position (GRCh38)	Frequency Brown (*n* = 46) ^1^	Frequency Blue (*n* = 19) ^1^	Variants in Strong LD ^2^ (r^2^ ≥ 0.8)
rs74409036	27,818,606	0.03	0.11	rs78114576
rs78544415	27,842,634	0.00	0.05	rs79071800, rs28565430, rs28423991, rs74474678 and rs80177321
rs72714116	28,083,061	0.00	0.08	rs72714116
rs191109490	28,120,403	0.00	0.05	rs543971307 and rs145048438
rs551217952	28,139,387	0.01	0.05	rs182498200 and rs184120129
rs62008729	28,173,140	0.01	0.08	rs62008729

^1^ Frequency of variant allele in 65 rs12913832 AA and AG individuals that did not have the missense variants rs74653330:T and rs121918166:T; ^2^ LD = Linkage disequilibrium.

**Table 3:** The frequencies of the six candidate variant alleles in the Norwegian biobank population.

			Variant Allele Frequency	
rs ID	Reference Allele	Variant Allele	Frequency Norway (*n* = 519)	Frequency Europe (GnomAD)
rs74409036	G	A	0.031	0.052
rs78544415	C	T	0.026	0.035
rs72714116	C	T	0.004	0.020
rs191109490	A	C	0.002	0.002
rs551217952	A	C	0.003	0.003
rs62008729	C	T	0.082	0.024

**Table 4:** *HERC2-OCA2* haplotypes estimated by PHASE.

Haplotypes ^a,c^	rs74409036:A	rs78544415:T	rs74653330:T	rs121918166:T	rs72714116:T	rs191109490:C	rs551217952:C	Frequency NOR ^b^ (*n* = 519)
A	-	-	-	-	-	-	-	0.135
G	-	-	-	-	-	-	-	0.772
G1	A	-	-	-	-	-	-	0.028
G2	-	T	-	-	-	-	-	0.025
G3	-	T	T	-	-	-	-	0.001
G4	-	-	-	T	-	-	-	0.001
A1	A	-	-	-	-	-	C	0.003
A3	-	-	T	-	-	-	-	0.024
A4	-	-	-	T	-	-	-	0.006
A5	-	-	-	-	T	-	-	0.004
A6	-	-	-	-	-	C	-	0.002

^a^ Haplotype A and G indicates the A-allele and G-allele in rs12913832, respectively; ^b^ NOR = Norwegian biobank population; ^c^ Reference allele.

## Data Availability

The data generated in the present study are included within the manuscript and supplementary files.

## Supplementary Materials

The following supporting information can be downloaded at: https://www.mdpi.com/article/10.3390/genes14030698/s1, Figure S1: PIE-scores and genotypes of rs121918166 and rs74653330 in the study cohort (*n* = 94); Figure S2: Chromosomal position of the six blue eye colour candidate variants (red), the two missense variants rs74653330 and rs121918166, and the main eye colour predictor SNP rs12913832. Pos = chromosomal position (GRCh38); Figure S3: Genotypes and PIE-scores for the candidate blue eye colour variants typed in rs12913832 AA and AG individuals (*n* = 165) from the Norwegian biobank population; Table S1: Genotype and eye colour information of the study population; Table S2: Information on primers used for SBE typing of candidate variants; Table S3: Variants that were statistically significantly associated with both PIE-score and eye colour categories (Kruskal–Wallis test and Fisher’s exact test, raw *p*-values ≤0.05) in rs12913832 AA and AG individuals.
