# Supplementary material for: Association between Variants in the OCA2-HERC2 Region and Blue Eye Colour in HERC2 rs12913832 AA and AG Individuals
Source: Genes (Basel). 2023 Mar 11;14(3):698. doi: 10.3390/genes14030698 (PMC10048254; doi:10.3390/genes14030698)
Supplement: Supplementary file 1 [file genes-14-00698-s001.zip › Supplement figures.pdf]

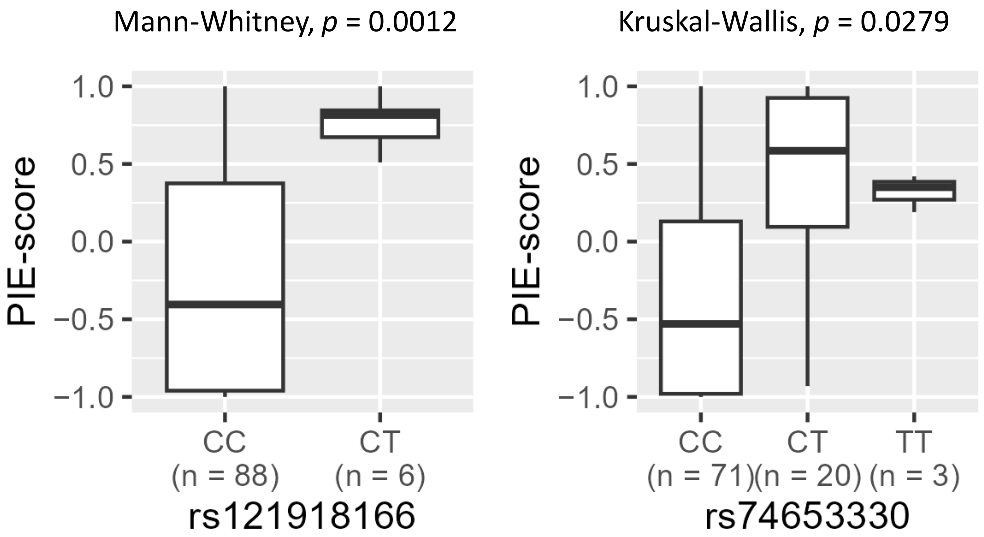

Figure S1: PIE scores and genotypes of rs121918166 and rs74653330 in the study cohort (*n* = 94).

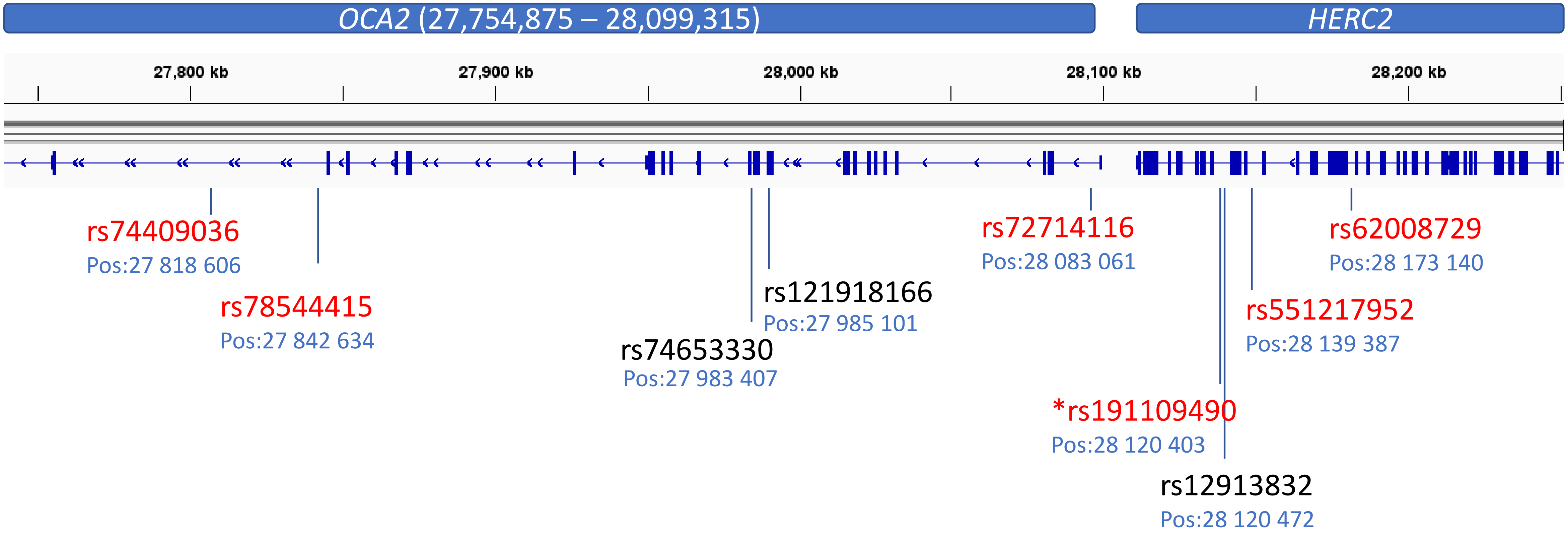

Figure S2: Chromosomal position of the six blue eye colour candidate variants (red), the two missense variants rs74653330 and rs121918166, and the main eye colour predictor SNP rs12913832. Pos = chromosomal position (GRCh38).

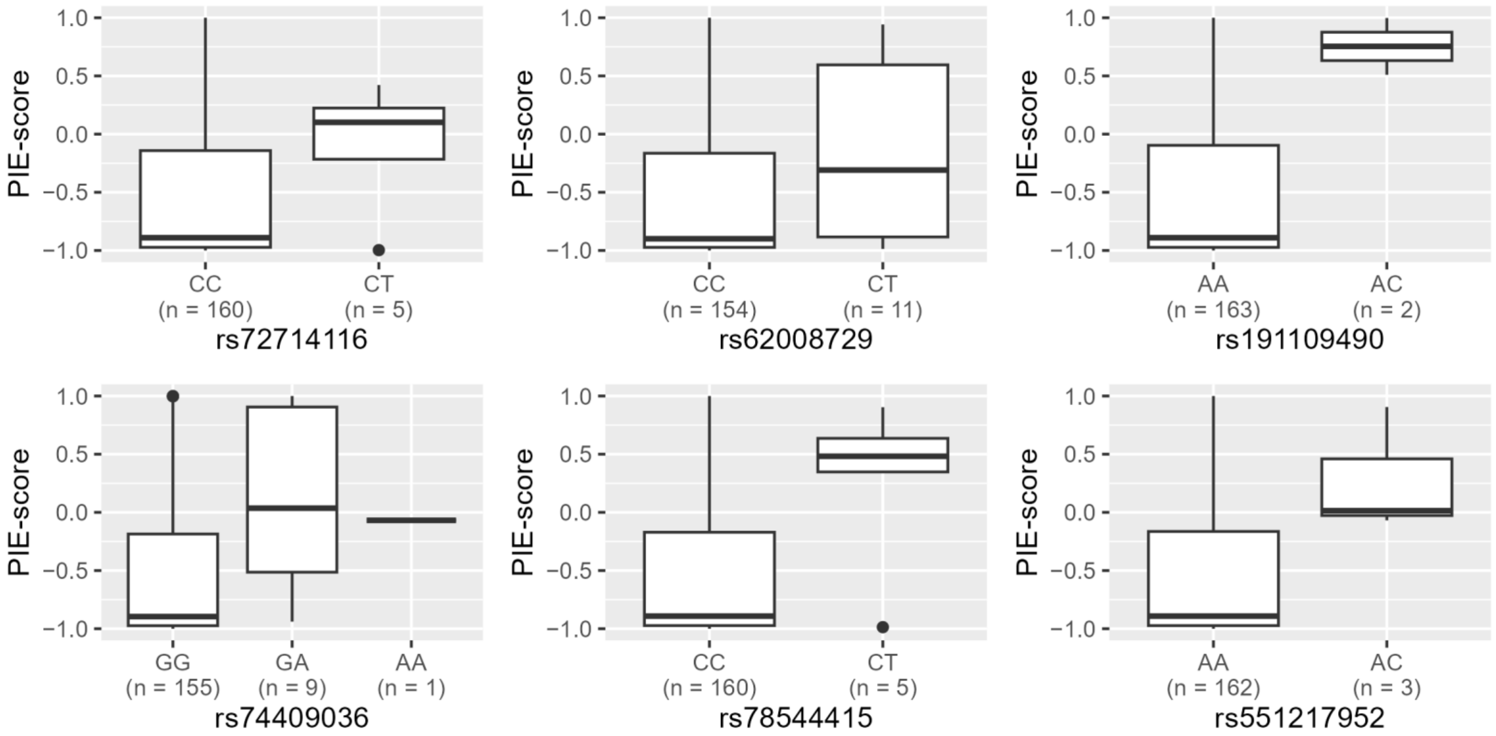

Figure S3: Genotypes and PIE-scores for the candidate blue eye colour variants typed in rs12913832 AA and AG individuals (*n* = 165) from the Norwegian biobank population.
